# Hemolysis During Ex Vivo Lung Perfusion Is Associated with Endothelial Injury, Cytokine Release, and Primary Graft Dysfunction

**DOI:** 10.3390/bioengineering13101230

**Published:** 2026-10-21

**Authors:** Chris H. H. Chan, Rupa Kanchi, Fernanda C. P. Mesquita, Patricia Da Costa Lopez, Yaxin Wang, Asma F. Farooqui, Wilson Lim, Andres Leon Pena, Emma E. Hills, Cristian Coarfa, Abdussalam Elsenousi, Aladdein Mattar, Nirmal S. Sharma, Ramiro Fernandez, Camila Hochman-Mendez, Gabriel Loor

**Affiliations:** 1 Innovative Device & Engineering Applications Lab, The Texas Heart Institute, Baylor College of Medicine, Houston, TX 77030, USA; yaxin.payne@bcm.edu (Y.W.); asma.farooqui@bcm.edu (A.F.F.); wilson.lim@bcm.edu (W.L.); 2 Advanced Technology Cores, Dan L Duncan Comprehensive Cancer Center, Baylor College of Medicine, Houston, TX 77030, USA; rupa.kanchi@bcm.edu (R.K.); patricialuciana.dacostalopez@bcm.edu (P.D.C.L.); coarfa@bcm.edu (C.C.); camila.hochmanmendez@bcm.edu (C.H.-M.); 3 Regenerative Medicine Research Lab, The Texas Heart Institute, Baylor College of Medicine, Houston, TX 77030, USA; fmesquita@texasheart.org; 4 Division of Cardiopulmonary Transplantation and Mechanical Circulatory Support, Michael E. DeBakey Department of Surgery, Baylor College of Medicine, Houston, TX 77030, USA; andres.leonpena@bcm.edu (A.L.P.); abdussalam.elsenousi@bcm.edu (A.E.); aladdein.mattar@bcm.edu (A.M.); nirmal.sharma@bcm.edu (N.S.S.); ramiro.fernandez@bcm.edu (R.F.)

**Keywords:** ex vivo lung perfusion, primary graft dysfunction, hemolysis, endothelial injury, cytokine release

## Abstract

Primary graft dysfunction (PGD) is a major cause of early morbidity and mortality after lung transplantation. During blood-based ex vivo lung perfusion (EVLP), hemolysis releases plasma-free hemoglobin (pfHb) and other erythrocyte-derived products that may promote endothelial injury and inflammation. We investigated whether EVLP-associated hemolysis is associated with cytokine release, endothelial dysfunction, and grade 3 PGD (PGD3). In this prospective, single-center observational study, end-EVLP perfusate from 57 double-lung transplant recipients supported with the Organ Care System Lung was analyzed for pfHb, 26 cytokines, blood counts, and associations with PGD3 within 72 h after transplantation. A mock EVLP blood-loop model was used to evaluate the effects of red blood cell storage duration and circuit run time on hemolysis. Generated hemolytic perfusates were applied to human lung microvascular endothelial cells to assess barrier injury and inflammatory activation. Among the 57 recipients, 32 (56.1%) developed PGD3 at any time point within 72 h after transplantation. Higher end-EVLP pfHb, interleukin-6, and interleukin-8 levels were associated with PGD3, and pfHb correlated positively with interleukin-6 and interleukin-8. Hemolysis increased with longer blood storage and EVLP duration, with storage duration emerging as the dominant contributor in the blood-loop model. Hemolytic perfusate disrupted endothelial junctions, increased permeability, and induced interleukin-6 and interleukin-8 release. EVLP-associated hemolysis is linked to endothelial barrier injury, inflammatory activation, and PGD3 after lung transplantation. Reducing hemolysis through improved blood-product selection and EVLP circuit management may represent a modifiable strategy to improve posttransplant outcomes.

## 1. Introduction

Ex vivo machine perfusion has emerged as an important strategy for donor-organ assessment, preservation, and recovery. In lung transplantation, ex vivo lung perfusion (EVLP) allows donor lungs to be reassessed under normothermic, ventilated, and perfused conditions and has supported expanded use of extended-criteria grafts [1,2,3,4,5]. Despite these advances, primary graft dysfunction (PGD) remains a major early complication after lung transplantation and is associated with morbidity, mortality, and poorer long-term graft survival [6]. EVLP-related biologic injury may contribute to the risk of PGD, particularly during blood-based or prolonged perfusion [7]. Importantly, EVLP provides a unique preimplantation window to evaluate graft injury, identify actionable biomarkers, and define modifiable processes that could improve posttransplant outcomes.

Blood-based EVLP perfusates provide physiologic oxygen-carrying capacity and support clinically relevant assessment of gas exchange [3,4,8,9]. However, exposure of red blood cells (RBCs) to supraphysiologic shear stress and turbulence within the blood pump, together with stasis and recirculation across artificial oxygenator surfaces, may impair erythrocyte deformability and promote hemolysis [10,11,12,13]. Hemolysis releases plasma-free hemoglobin (pfHb), heme, and iron, which can promote nitric oxide scavenging, oxidative stress, endothelial activation, vasoconstriction, and microvascular barrier dysfunction [14,15,16]. These mechanisms are particularly relevant in the pulmonary circulation, where endothelial injury and increased permeability are central to edema formation, ischemia–reperfusion injury, and PGD.

Hemolysis may also amplify inflammatory signaling. pfHb and related hemolysis products have been linked to thromboinflammation and release of cytokines such as interleukin (IL)-6 and IL-8, both of which contribute to endothelial injury and neutrophil recruitment in acute lung injury [17,18]. Other inflammatory mediators may also contribute to blood-cell injury; for example, TNF-α has been shown to induce apoptosis in K562 hematopoietic erythroleukemia cells, further supporting the potential role of inflammatory signaling in hematopoietic cell damage [19]. Although hemolysis, endothelial injury, and inflammatory activation have each been implicated in lung injury, their interrelationship during clinical EVLP and their association with subsequent PGD remain incompletely defined. It is also unclear whether hemolysis during blood-based EVLP is driven primarily by circuit exposure time, blood storage duration, or both.

We therefore performed a translational study combining clinical EVLP perfusate biomarker analysis, an EVLP-like blood-loop model, and in vitro HLMVEC experiments. We tested the hypotheses that (1) higher end-EVLP pfHb and inflammatory cytokines are associated with PGD3 that arises within 72 h after transplantation; (2) older stored blood increases susceptibility to EVLP-associated hemolysis; and (3) hemolytic perfusate directly injures lung microvascular endothelium by disrupting vascular endothelial cadherin (VE-cadherin) junctions, increasing permeability, and inducing IL-6/IL-8 release.

## 2. Materials and Methods

### 2.1. Study Design and Clinical Cohort

This prospective, observational, single-center study included 57 double-lung transplant recipients treated at Baylor St. Luke’s Medical Center between 11 March 2018 and 6 December 2023. The study was approved by the Baylor College of Medicine Institutional Review Board (IRB H-42256), and all patients provided informed consent for clinical data collection, sample collection, and specimen storage. Clinical data were obtained from the Baylor St. Luke’s Medical Center lung transplant database.

The primary objective was to compare end-EVLP perfusate hemolysis and 26 cytokine biomarkers between recipients who developed grade 3 primary graft dysfunction (PGD3) within 72 h after transplantation and those who did not. The clinical component was observational, as no experimental intervention was applied to transplanted lungs beyond standard EVLP and transplantation practice.

This study was designed as an early-stage translational biomarker and mechanism-focused analysis. Clinical perfusate measurements were used to identify associations between EVLP injury markers and PGD3, while controlled in vitro blood-loop and endothelial cell experiments were performed to evaluate biologically plausible mechanisms of blood trauma and pulmonary endothelial injury. These experimental components were intended to support mechanistic interpretation rather than to establish definitive clinical causality.

### 2.2. PGD Grading and Outcomes

Perioperative chest radiographs and blood gas results were reviewed by an expert PGD grader. PGD was identified according to the 2016 International Society for Heart and Lung Transplantation consensus guidelines, which included using the oxygen saturation-based scale when arterial blood gas measurements were unavailable [20,21]. The primary outcome was PGD3, defined as PaO_2_/FiO_2_ < 200 with compatible radiographic findings at any time within 72 h after transplantation. Posttransplant outcomes, including hospital length of stay, duration of ventilatory support, airway complications, tracheostomy, and survival at 3 and 12 months, were recorded.

### 2.3. Clinical EVLP Protocol and Perfusate Collection

EVLP was performed using the portable Organ Care System (OCS) Lung (TransMedics Inc., Andover, MA, USA) according to established clinical protocols [3,4]. Donor lungs were flushed antegrade and retrograde with cold buffered OCS Lung Solution containing nitroglycerin, connected to the OCS Lung, gradually warmed to 37 °C, and ventilated with standard settings. The EVLP perfusate consisted of 3 units of packed RBCs (O-positive or O-negative) and 1.5 L of OCS Lung Solution, with a target hematocrit of 15–25%. Perfusion hemodynamics and PaO_2_/FiO_2_ were assessed during EVLP and before final acceptance for transplantation.

At the end of EVLP, 10 mL of blood-based perfusate was collected into ethylenediaminetetraacetic acid (EDTA) tubes and transported immediately to The Texas Heart Institute Biorepository and Biospecimen Profiling Core. Complete blood counts were measured using a KX-21N hematology analyzer (Sysmex Corporation, Kobe, Japan). Plasma was isolated by centrifugation at 400× *g* for 20 min at 4 °C, aliquoted, stored at −80 °C, and later analyzed for multiplex cytokines and hemolysis.

Blood storage duration was abstracted from blood-bank records for 53 of 57 patients; data were missing for the other 4 patients. EVLP duration was defined as the time from initiation of OCS Lung perfusion to final perfusate sampling. These variables were analyzed in both clinical and experimental settings because they represent practical, modifiable components of the blood-based EVLP workflow.

### 2.4. Cytokine and Hemolysis Assays

Serum cytokine concentrations were measured using the Bio-Plex Pro™ Human Cytokine 27-plex Assay (M500KCAF0Y; Bio-Rad Laboratories, Hercules, CA, USA) according to the manufacturer’s instructions. This multiplex bead-based assay enables the simultaneous quantification of multiple cytokines, chemokines, and growth factors from serum samples. Cytokine concentrations were measured using the Luminex INTELLIFLEX instrument (Luminex Corporation, Austin, TX, USA) and calculated from standard curves generated for each analyte. The methodology for multiplex cytokine profiling is consistent with previously described approaches for comprehensive cytokine analysis in biological samples [22]. A coefficient of variation < 20% was required for assay acceptance. pfHb was measured in duplicate using the Harboe assay after dilution in 0.1% Na_2_CO_3_. Absorbance was measured at 380, 415, and 450 nm, and pfHb was calculated as: (167.2 × A_415_) − (83.6 × A_380_) − (83.6 × A_450_) [23].

### 2.5. Mock EVLP Blood-Loop Model

**Figure 1:**
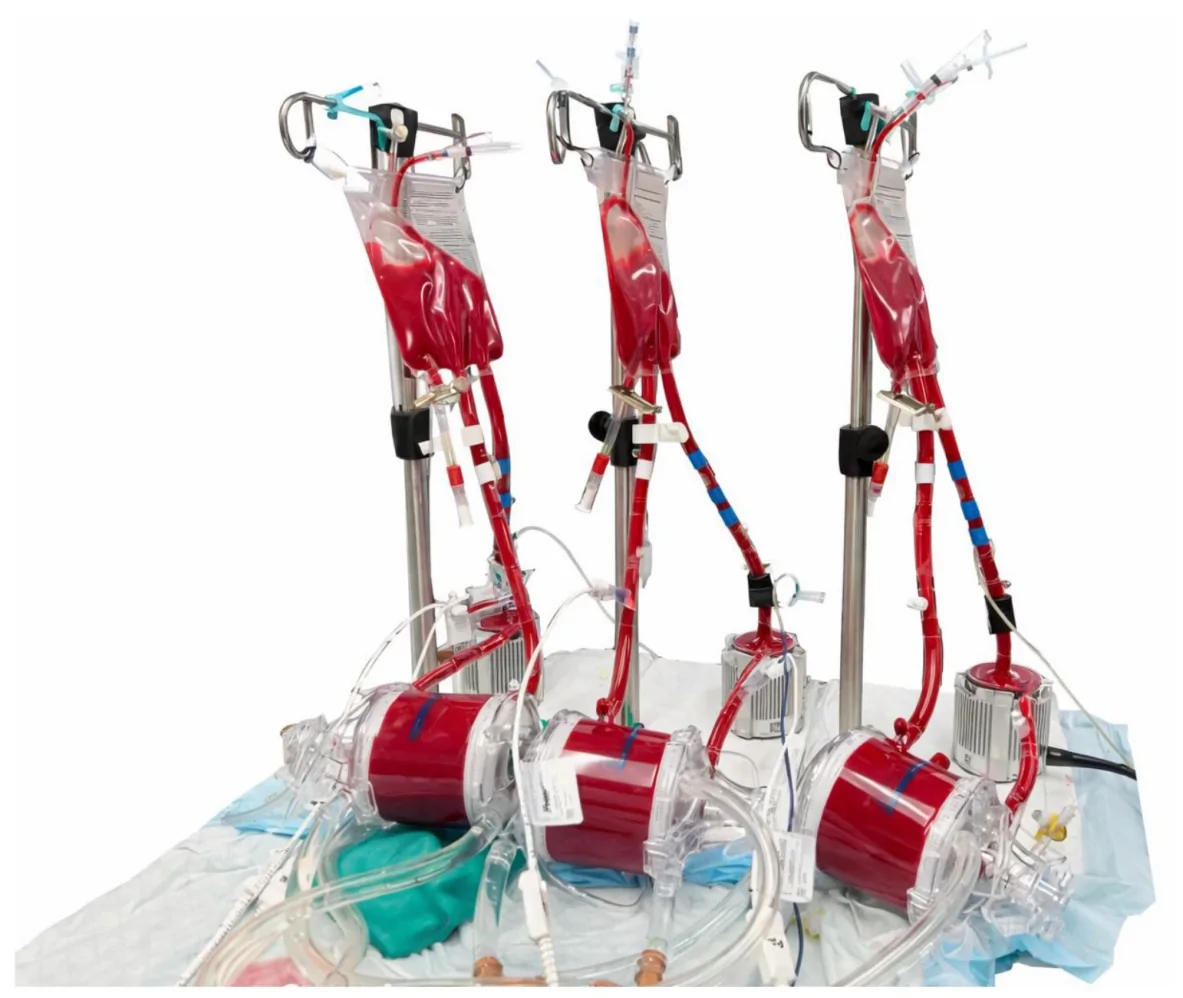
Standardized in vitro blood-loop circuit for evaluating the independent effects of blood storage duration and circuit run time on hemolysis under EVLP-like hemodynamic conditions. Three identical CentriMag-based circuits were used to assess the independent effects of blood storage duration and EVLP-like circuit run time on hemolysis under EVLP-like hemodynamic conditions. Human blood stored for 1, 3, or 5 weeks was perfused at 2.5 L/min with a 30- to 35-mmHg oxygenator pressure drop at 37 °C for 24 h. Samples were collected at prespecified time points for plasma-free hemoglobin evaluation, complete blood count, and blood gas analyses.

Three identical in vitro blood-loop circuits were used to evaluate the independent effects of circuit run time and blood storage duration (Figure 1). Four human blood bags were obtained from the Gulf Coast Regional Blood Center and tested at storage weeks 1, 3, and 5. Blood was circulated using CentriMag centrifugal pumps (Thoratec Corporation, an Abbott company, Pleasanton, CA, USA) under EVLP-like conditions: 1500 rpm, 2.5 L/min flow, 30- to 35-mmHg oxygenator pressure differential, 37 ± 0.3 °C, and pH 7.4 ± 0.3. Each circuit contained 300 mL total volume, consisting of 120 mL blood and 180 mL Perfadex, yielding a hematocrit of 17.4% ± 0.6%. Samples were collected at 0, 3, 6, 9, 12, 21, and 24 h for pfHb, complete blood count, and blood gas analysis.

### 2.6. Endothelial Cell Injury, VE-Cadherin, Permeability, and Cytokine Release

**Figure 2:**
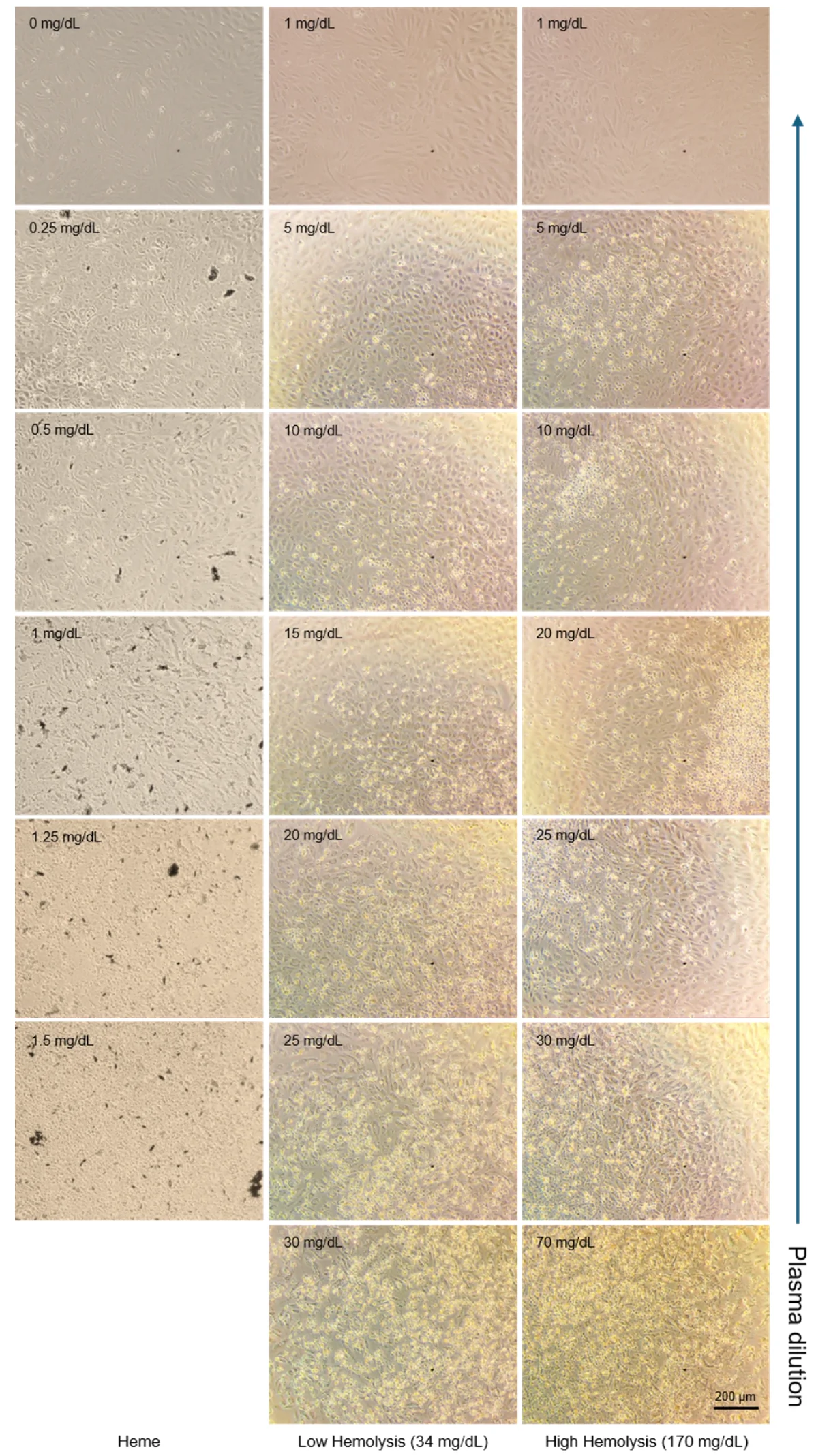
Dose-ranging assessment of hemolytic perfusate exposure in human lung microvascular endothelial cells. These cells were exposed for 16 h to increasing concentrations of plasma-free hemoglobin (pfHb)-containing perfusate to identify exposure conditions that induced endothelial injury while preserving sufficient cell attachment for mechanistic analysis. Representative phase-contrast images show progressive morphologic changes with increasing hemolysis. Lower pfHb concentration produced minimal effects, whereas the highest doses induced marked cellular injury and cell death.

HLMVECs were cultured under standard conditions and exposed for 16 h to heme or hemolytic perfusate generated from the blood-loop model. Hemolytic dose-ranging studies were performed to identify exposure conditions that induced endothelial stress while preserving sufficient cell attachment for downstream analyses (Figure 2). VE-cadherin organization was assessed by immunofluorescence using an anti-VE-cadherin intercellular junction marker antibody (Cat. No. ab33168; Abcam, Cambridge, UK), Alexa Fluor™ 488-conjugated goat antirabbit IgG (H + L) cross-adsorbed secondary antibody (Cat. No. A-11008; Invitrogen, Thermo Fisher Scientific, Carlsbad, CA, USA), and 4′,6-diamidino-2-phenylindole (DAPI) counterstaining (DAPI; Invitrogen, Thermo Fisher Scientific, Carlsbad, CA, USA). Endothelial permeability was measured using fluorescein isothiocyanate (FITC)-dextran 70 (Cat. No. 46945; Sigma-Aldrich, St. Louis, MO, USA) across confluent cell-culture inserts, with fluorescence quantified over 180 min. IL-6 and IL-8 concentrations in cell-culture supernatants and blood-loop plasma were measured using a multiplex immunoassay (Cat. No. HSTCMAG-28SK-02; MilliporeSigma, Burlington, MA, USA) on an xMAP INTELLIFLEX instrument (Luminex Corporation, Austin, TX, USA).

### 2.7. Statistical Analysis

Cytokine and pfHb data from 57 clinical samples were log_2_-transformed and batch-corrected using removeBatchEffect() from the R limma package. Differential expression between PGD3 and non-PGD3 groups was assessed using *t* tests with Benjamini–Hochberg false discovery rate adjustment; a false discovery rate < 0.25 was considered significant for exploratory biomarker discovery. Spearman correlation assessed associations between pfHb and cytokines. Clinical associations of blood storage duration and EVLP duration with hemolysis were assessed by 1-way analysis of variance (ANOVA) with Tukey multiple-comparison testing. Blood-loop data were analyzed using repeated-measures mixed-effects models with blood bag as a random effect. Analyses were performed in R version 4.4.3 (R Foundation for Statistical Computing, Vienna, Austria) and GraphPad Prism 10.6.1 (GraphPad Software, LLC, Boston, MA, USA).

## 3. Results

### 3.1. Clinical Outcomes by PGD3 Status

Among 57 double-lung transplant recipients, 32 (56.1%) developed PGD3 at any time point within 72 h after transplantation (Table 1). Recipient and donor characteristics were generally comparable between PGD3 and non-PGD3 groups, including recipient age, body mass index, primary diagnosis, donor age, donor type, last donor PaO_2_/FiO_2_, EVLP duration, and total out-of-body time. Despite similar baseline characteristics, recipients who developed PGD3 experienced substantially worse early posttransplant outcomes, including longer median hospital length of stay (29 vs. 19 days; *p* = 0.013), ventilation for 5 days or longer (69% vs. 28%; *p* < 0.001), and tracheostomy (53% vs. 20%; *p* = 0.011). One-year mortality was higher in the PGD3 group (25% vs. 0%; *p* = 0.007), and 90-day mortality showed a similar trend (16% vs. 0%; *p* = 0.061).

### 3.2. End-EVLP pfHb and Inflammatory Biomarkers Were Associated with PGD3

**Figure 3:**
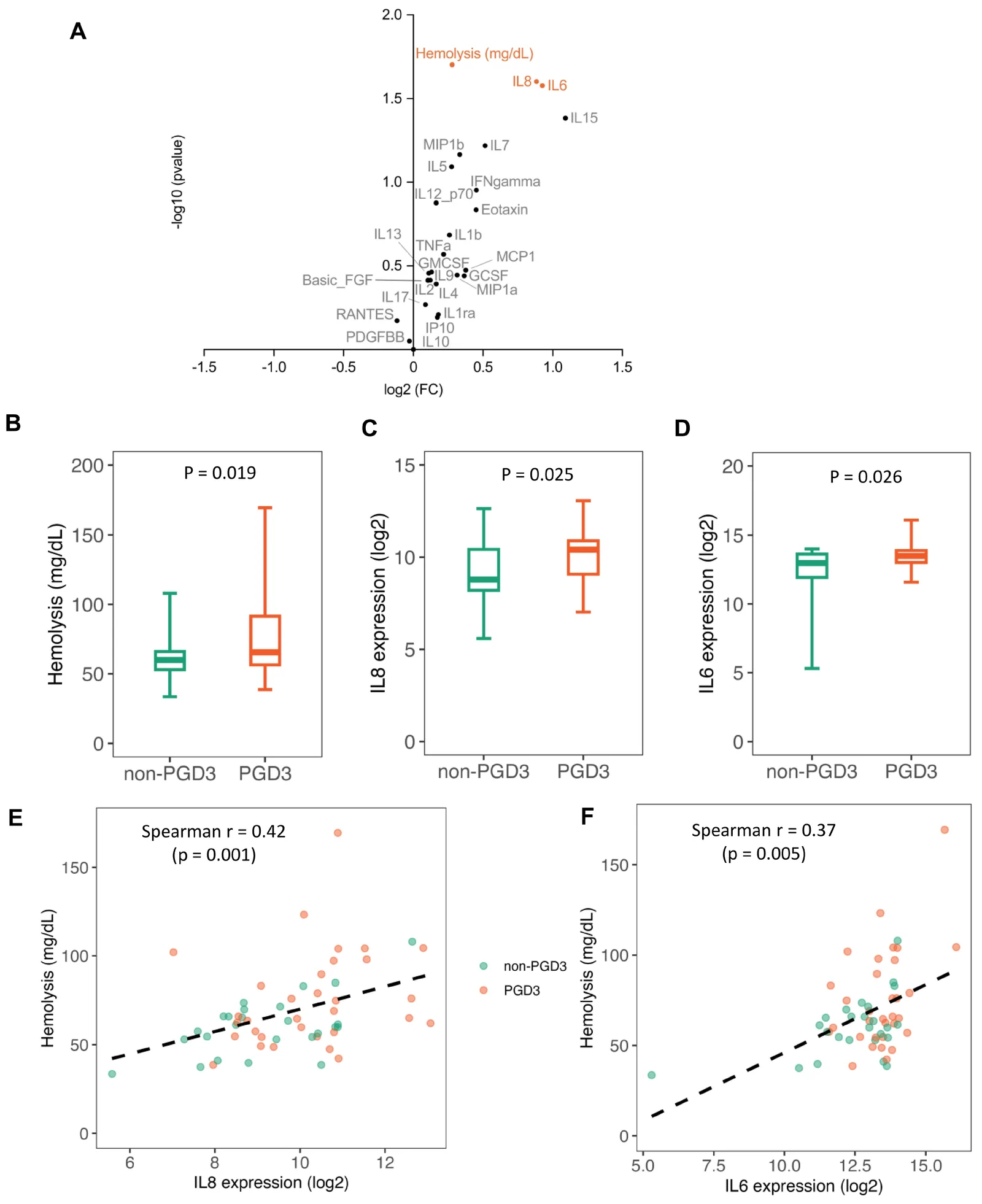
Batch-corrected hemolysis and cytokine analyses comparing recipients with and without PGD3 within 72 h after transplantation. End-EVLP perfusate samples were analyzed for plasma-free hemoglobin (pfHb) and 26 inflammatory cytokines, then stratified by the presence or absence of grade 3 primary graft dysfunction (PGD3) within 72 h after transplantation (**A**). Recipients who developed PGD3 had higher end-EVLP pfHb (**B**), interleukin (IL)-8 (**C**), and IL-6 (**D**) levels than recipients without PGD3. Correlation analysis revealed significant positive associations between pfHb and IL-8 (**E**) and between pfHb and IL-6 (**F**), supporting a relationship between EVLP-associated hemolysis, inflammatory activation, and early graft dysfunction.

To identify EVLP-associated injury markers linked to subsequent graft dysfunction, end-EVLP perfusate samples were analyzed for pfHb and 26 cytokines (Figure 3A). After batch correction and multiple-testing adjustment, pfHb, IL-6, and IL-8 emerged as the only biomarkers significantly associated with PGD3 within 72 h after transplantation (false discovery rate < 0.25). Recipients who developed PGD3 had higher end-EVLP pfHb, IL-6, and IL-8 levels than recipients without PGD3 (Figure 3B–D). Correlation analysis showed a positive association between pfHb and IL-8 (Spearman r = 0.42; *p* = 0.001; Figure 3E) and between pfHb and IL-6 (Spearman r = 0.37; *p* = 0.005; Figure 3F). These findings suggest a relationship between EVLP-associated hemolysis and inflammatory activation before implantation.

In contrast, complete blood count parameters, including white blood cell count, RBC count, platelet count, hemoglobin, and hematocrit, did not differ significantly between groups (Table 2), indicating that the observed biomarker differences were not explained by broad variations in perfusate cellular composition.

### 3.3. Longer Blood Storage Duration and EVLP Duration Are Associated with Greater Hemolysis in Clinical EVLP

**Figure 4:**
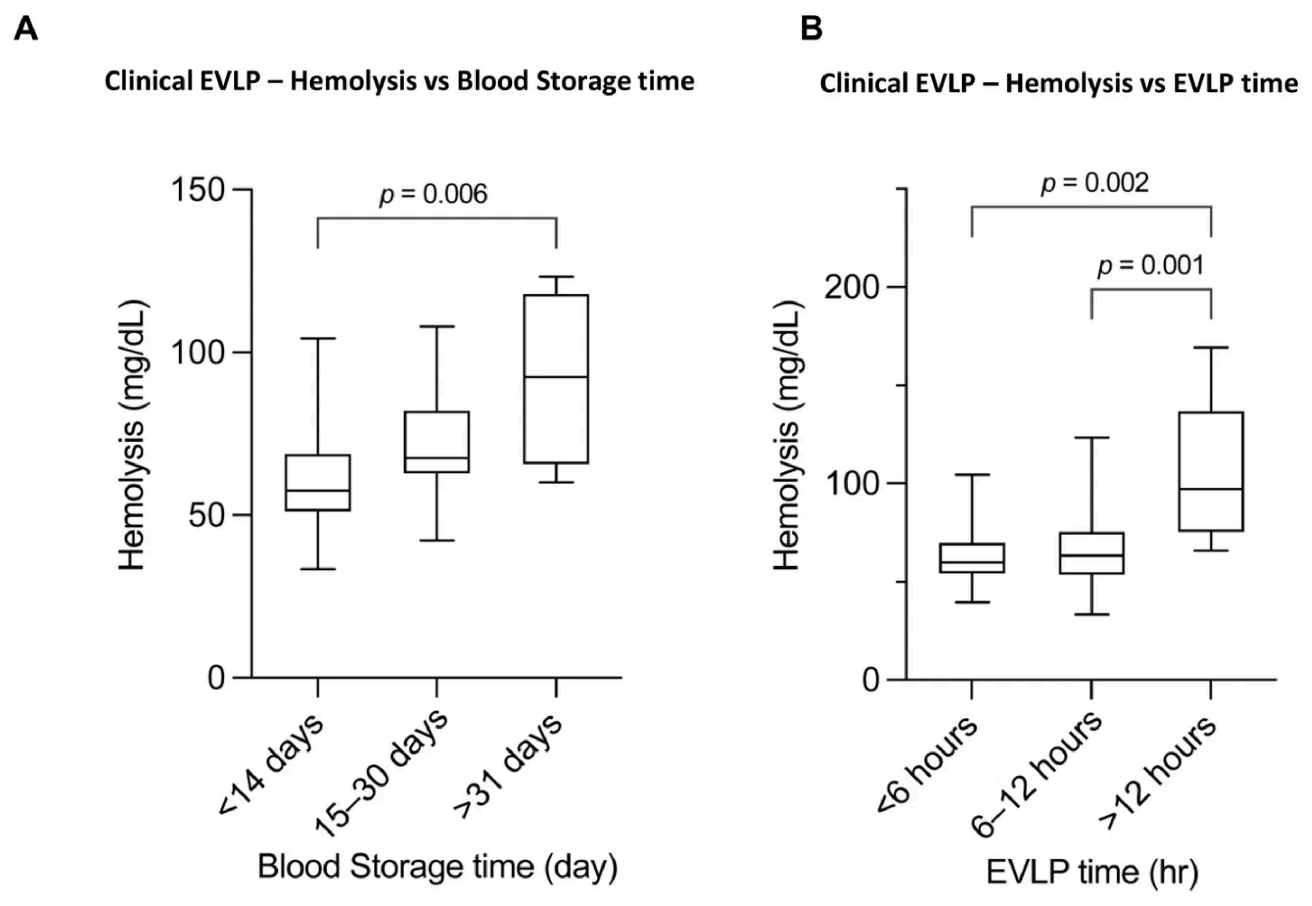
Effects of blood storage duration and EVLP duration on perfusate hemolysis in the clinical EVLP cohort. Plasma-free hemoglobin levels were compared by blood storage duration (**A**) and EVLP duration (**B**). Longer blood storage and prolonged EVLP were associated with greater perfusate hemolysis, suggesting contributions from both red blood cell storage injury and extended circuit perfusion.

Given the association between hemolysis and PGD3, we next evaluated potential clinical determinants of perfusate hemolysis in the EVLP cohort (Figure 4). Perfusate pfHb levels increased with longer RBC storage duration and were significantly greater when perfusates were prepared with blood stored for >31 days compared with <14 days (*p* = 0.006; Figure 4A). Perfusate pfHb concentrations also increased with longer EVLP duration. Lungs perfused for >12 h had significantly higher pfHb levels than those perfused for <6 h (*p* = 0.002) or 6–12 h (*p* = 0.001; Figure 4B). These findings suggest that both RBC storage age and prolonged circuit exposure are associated with greater hemolysis during clinical EVLP.

### 3.4. Blood Storage Duration Is the Primary Determinant of Hemolysis Under Controlled EVLP-like Conditions

To independently evaluate the contributions of blood storage duration and circuit run time to hemolysis, we employed a standardized mock EVLP blood-loop model (Figure 1). Across 24 h of perfusion, hemolysis differed significantly according to blood storage duration (*p* = 0.004), whereas the effect of circuit run time alone did not reach statistical significance (*p* = 0.067).

**Figure 5:**
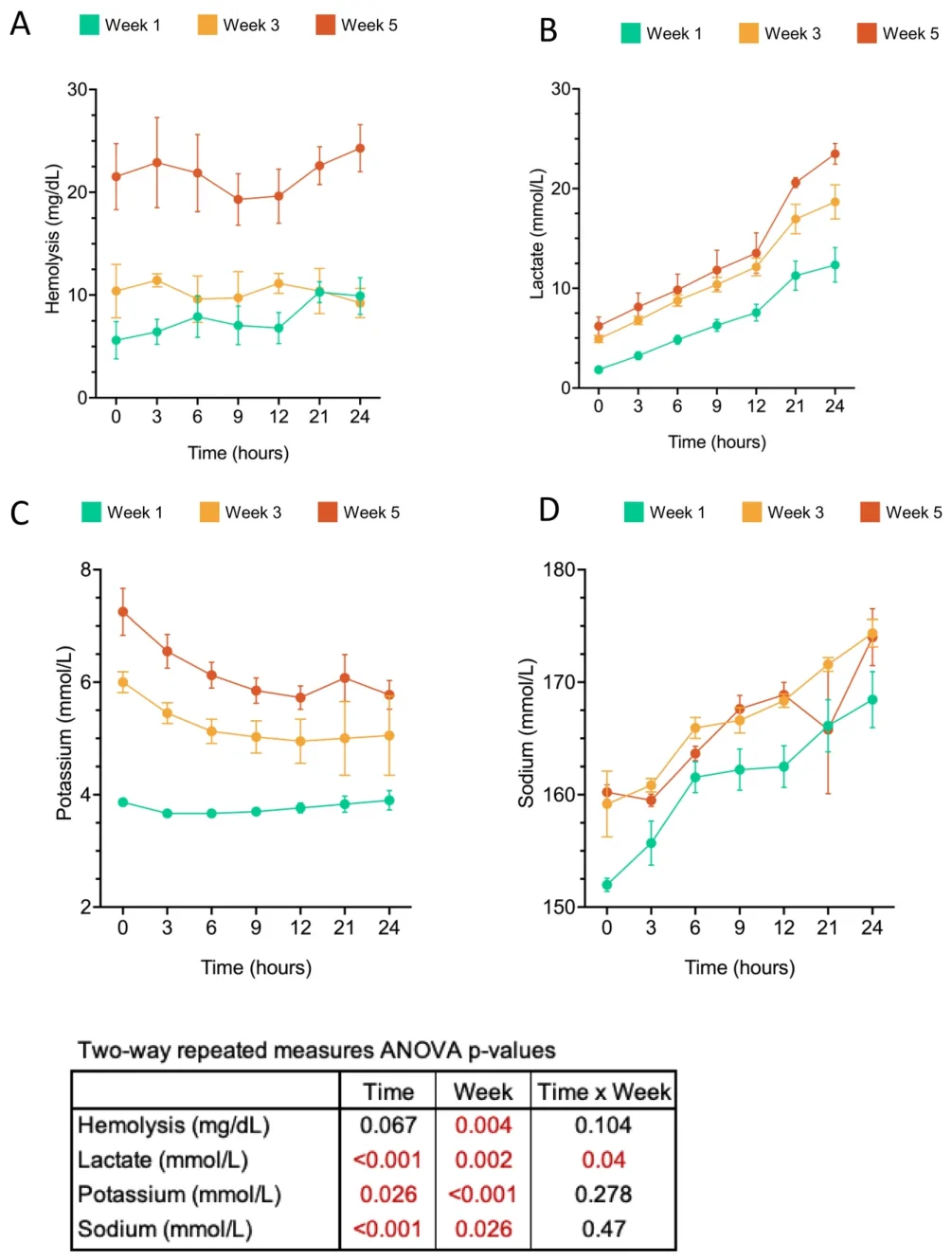
Effects of blood storage duration and EVLP-like circuit run time on hemolysis and metabolic buildup. Under standardized EVLP-like conditions, 3 identical in vitro blood-loop circuits were perfused for 24 h with human blood stored for 1, 3, or 5 weeks. (**A**) Week 5 blood showed the most hemolysis, consistent with increased red blood cell fragility after prolonged storage. (**B**) Lactate concentration increased over 24 h, with greater accumulation in older blood. (**C**) Potassium level was highest in week-5 blood, consistent with storage-related electrolyte changes. (**D**) Sodium concentration increased gradually, with similar trends across groups. ANOVA, analysis of variance.

Week 5 blood consistently generated higher pfHb levels than week 1 and week 3 blood throughout the experiment (Figure 5A), indicating greater susceptibility of older stored blood to mechanical injury under EVLP-like conditions. In contrast, circuit run time had a greater effect on metabolic parameters. Lactate accumulation increased with both blood storage duration (*p* < 0.001) and perfusion time (*p* = 0.002). Potassium and sodium levels also changed over time and with blood age (Figure 5B–D).

These findings suggest that blood storage duration is a primary determinant of hemolysis under controlled EVLP-like conditions. Ongoing circuit perfusion, in contrast, contributes predominantly to metabolic and electrolyte alterations.

### 3.5. Hemolytic Perfusate Induces Endothelial Injury and Inflammatory Activation

**Figure 6:**
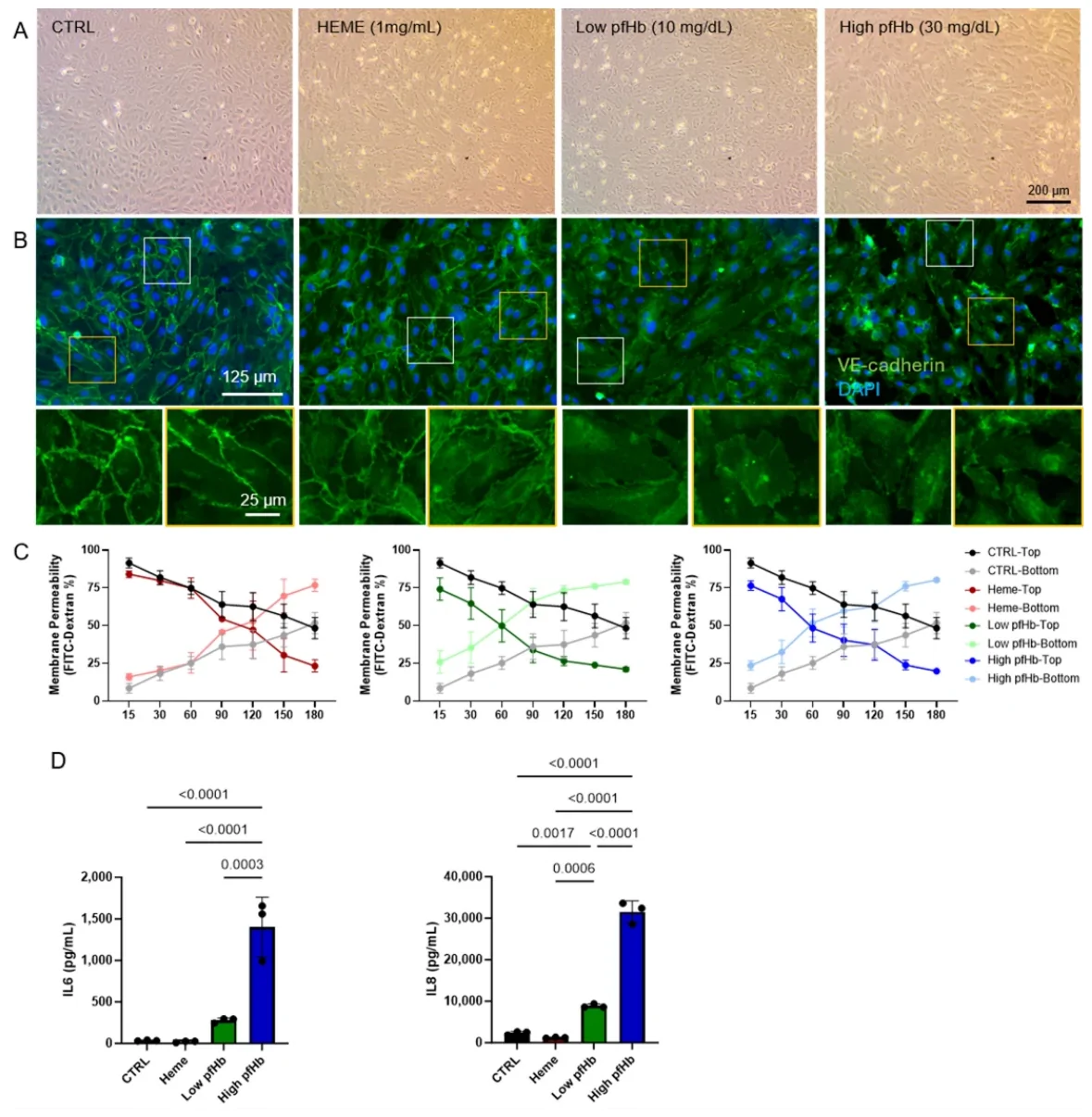
In vitro assessment of hemolysis-induced endothelial injury and inflammatory activation. Human lung microvascular endothelial cells were exposed to increasing levels of hemolysis to evaluate endothelial barrier injury and cytokine release. (**A**) Representative phase-contrast images show progressive morphologic changes with increasing hemolysis. (**B**) Vascular endothelial cadherin (VE-cadherin) immunofluorescence shows dose-dependent disruption of junctional integrity. (**C**) Endothelial permeability increased in a hemolysis-dependent manner. (**D**) Enzyme-linked immunosorbent assay (ELISA) analysis showed increased endothelial interleukin (IL)-6 and IL-8 release after hemolytic exposure, supporting a direct link between hemolysis, endothelial activation, and inflammatory signaling. CTRL, control; DAPI, 4′,6-diamidino-2-phenylindole; FITC, fluorescein isothiocyanate; HEME, pfHb, plasma-free hemoglobin.

To evaluate the biologic consequences of EVLP-associated hemolysis, HLMVECs were exposed to hemolytic perfusate generated in the in vitro blood-loop model (Figure 6A). Hemolytic exposure produced progressive morphologic evidence of endothelial injury, including cellular stress, altered cell morphology, increased intercellular spacing, and accumulation of dark cytoplasmic granules. Consistent with endothelial barrier disruption, VE-cadherin staining revealed disruption of adherens junction organization after hemolytic exposure. Control cells showed continuous junctional VE-cadherin, whereas heme and hemolytic perfusate reduced membrane-associated staining and increased discontinuity of cell–cell junctions, with the greatest disruption observed under high-hemolysis conditions (Figure 6B).

Functional consequences of junctional injury were confirmed by permeability studies. Hemolytic exposure accelerated FITC-dextran transfer across endothelial monolayers in a dose-dependent manner, with low- and high-hemolysis conditions exhibiting earlier top-to-bottom tracer exchange than heme-treated and control groups (Figure 6C).

Hemolytic perfusate also induced robust endothelial inflammatory activation. IL-8 concentrations increased to 8842 ± 461 pg/mL in the low-hemolysis group and 31,524 ± 2644 pg/mL in the high-hemolysis group, whereas IL-6 increased to 1401 ± 360 pg/mL under high-hemolysis conditions (Figure 6D). In contrast, IL-6 and IL-8 were not detected in plasma collected directly from the in vitro blood-loop circuit in the absence of endothelial cells, supporting endothelial cells as the source of hemolysis-induced cytokine production.

Together, these findings show that hemolytic perfusate directly disrupts endothelial barrier integrity and induces inflammatory cytokine release, providing biologic plausibility for the association between EVLP-associated hemolysis, inflammatory activation, and PGD3 observed in the clinical cohort.

## 4. Discussion

This translational study identified EVLP-associated hemolysis as a potential contributor to endothelial injury, inflammatory activation, and PGD3 after lung transplantation. In 57 double-lung transplant recipients supported with blood-based EVLP, elevated end-EVLP pfHb, IL-6, and IL-8 were associated with PGD3 within 72 h and with worse clinical outcomes, including longer hospitalization, prolonged ventilation, increased tracheostomy, and higher 1-year mortality. Mechanistic studies showed that hemolytic perfusate disrupted VE-cadherin organization, increased endothelial permeability, and induced IL-6/IL-8 release in HLMVECs. Together, these findings support a model in which EVLP-associated hemolysis is linked to inflammatory and endothelial dysfunction. Importantly, EVLP provides a unique opportunity to assess graft injury before implantation, when injury processes may still be modifiable. Perfusate pfHb, IL-6, and IL-8 may therefore serve as markers of graft injury, indicators of EVLP process quality, and potential therapeutic targets to reduce downstream graft dysfunction.

These findings underscore the importance of hemocompatibility in EVLP platform design. While cellular perfusates provide oxygen-carrying capacity for prolonged support and may improve physiologic graft assessment, they also increase the risk of RBC trauma during extended extracorporeal circulation [24,25,26]. Supraphysiologic shear stress, artificial surfaces, recirculation, stasis, turbulence, and gas-exchange membranes may all contribute to erythrocyte injury [27,28,29]. Once hemolysis occurs, pfHb and heme can scavenge nitric oxide, increase oxidative stress, disrupt endothelial barrier integrity, and promote pulmonary vascular dysfunction [14,15,16,30,31,32].

The endothelial cell data provide mechanistic support for this interpretation. VE-cadherin is essential for adherens junction integrity and pulmonary microvascular barrier function. Hemolytic perfusate reduces junctional VE-cadherin, increases intercellular gap formation, accelerates FITC-dextran permeability, and induces IL-8 and IL-6 release [33]. The robust IL-6 and IL-8 responses are particularly notable, as both cytokines play important roles in neutrophil recruitment and inflammatory signaling and were also positively correlated with pfHb levels in clinical perfusate samples [34]. Heme alone produced some features of endothelial injury but did not reproduce the magnitude of inflammatory activation induced by hemolytic perfusate, suggesting that multiple hemolysis-associated mediators probably contribute to endothelial activation during blood-based EVLP.

Blood storage duration emerged as a potentially modifiable contributor to hemolysis. In the clinical cohort, hemolysis was greater when blood was stored for >31 days and when EVLP duration exceeded 12 h. In the controlled blood-loop model, storage duration was the dominant determinant of pfHb accumulation, whereas 24-h circuit run time had a weaker effect. These findings are consistent with RBC storage lesions, including reduced deformability, increased membrane fragility, electrolyte shifts, and metabolic deterioration [35,36]. Notably, despite significant differences in hemolysis between the PGD and non-PGD groups, total hemoglobin concentrations and hematocrit levels remained comparable in the perfusate at the end of EVLP. This finding suggests that even minor erythrocyte membrane disruption can substantially increase pfHb levels without producing detectable changes in total erythrocyte mass. EVLP programs could therefore benefit from incorporating blood-quality criteria such as storage duration, baseline pfHb, potassium, lactate, hematocrit, and potentially RBC deformability.

The apparent difference between the clinical and blood-loop findings is informative. In clinical EVLP, longer run time and older blood may coexist, and injured donor lungs may contribute inflammatory, metabolic, or endothelial factors that are absent from an isolated circuit model. The blood-loop data suggest that circuit exposure alone, under controlled conditions, is not sufficient to reproduce the full clinical hemolysis burden. Instead, blood quality before EVLP appears to determine the vulnerability of RBCs to subsequent mechanical stress.

Several strategies could reduce hemolysis-related EVLP injury. These include preferential use of fresher blood when feasible, evaluation of donor-derived or autologous blood sources, continuous monitoring of pfHb and perfusate chemistry, improved circuit hemocompatibility, reduced shear stress, optimized pump and oxygenator selection, minimized surface interactions, and therapies that protect endothelium or neutralize pfHb-induced injury [33,37,38]. These strategies are especially relevant as EVLP evolves from short-term assessment toward prolonged preservation and therapeutic organ recovery. The findings also support the use of end-EVLP perfusate biomarkers as indicators of EVLP process quality. pfHb is inexpensive, is readily measurable, and directly reflects RBC injury, whereas IL-6 and IL-8 levels may provide complementary information about endothelial and inflammatory activation. In addition to conventional hemolysis markers, lactate dehydrogenase (LDH) may serve as a sensitive indicator of erythrocyte membrane injury, as disruption of the red blood cell membrane results in the release of intracellular LDH into the circulation [39]. Because these biomarkers can be assessed before implantation, they may offer advantages over traditional posttransplant indicators of graft dysfunction. Serial monitoring during EVLP could potentially identify lungs at elevated risk for inflammatory injury, guide corrective interventions, or inform transplant decision-making. Future studies should identify clinically meaningful hemolysis thresholds and determine whether interventions that reduce hemolysis translate into lower PGD incidence and better graft outcomes.

### Limitations

This study has limitations. It was single-center and had a modest sample size, limiting multivariable adjustment and subgroup analysis. End-EVLP cytokine measurements provide only a terminal snapshot and do not capture temporal biomarker changes. Serial measurements across multiple stages would better characterize cytokine kinetics during EVLP. The endothelial cell model isolates direct endothelial responses but does not reproduce the full immune, mechanical, and reperfusion environment of transplanted lungs. The blood-loop model replicated key hemodynamic conditions but cannot model interactions between pulmonary tissue, perfusate, and the transplant recipient. Finally, the clinical data support association and biologic plausibility but do not prove causality between hemolysis and PGD3.

Additional work is needed to validate these findings in larger, multicenter EVLP cohorts and to determine whether hemolysis reduction changes clinical outcomes. Reticulocyte counts were not available in the present study and should be considered in future investigations where appropriate. Future studies should include serial perfusate sampling, standardized blood-product quality assessment, harmonized PGD adjudication, and integration of endothelial injury biomarkers with functional EVLP parameters such as oxygenation, compliance, pulmonary vascular resistance, and lung weight gain. Such studies would clarify whether pfHb and related cytokines can serve as actionable biomarkers for EVLP quality control.

## 5. Conclusions

In conclusion, end-EVLP hemolysis, together with elevated IL-6 and IL-8, was associated with PGD3 and poorer posttransplant outcomes. Hemolytic perfusate directly disrupted lung microvascular endothelial junctions, increased permeability, and induced inflammatory cytokine release. Prolonged blood storage was a major contributor to hemolysis under EVLP-like conditions. These findings support further investigation of blood storage practices, hemocompatible circuit design, and real-time hemolysis monitoring as potential strategies to reduce EVLP-associated injury and improve posttransplant outcomes.

## Figures and Tables

**Table 1:** Recipient and donor characteristics, ex vivo lung perfusion data, and posttransplant outcomes stratified by PGD3 status within 72 h after lung transplantation.

Variable	No PGD3 *n* = 25	PGD3 Within 72 h *n* = 32	*p* Value
**Baseline characteristics**			
Recipient age, y	58 (55, 63)	60 (39, 66)	0.9
Recipient sex			0.7
Male	14 (56)	16 (50)	
Female	11 (44)	16 (50)	
Body mass index	25.0 (21.1, 30.3)	27.5 (21.1, 30.3)	0.8
Primary diagnosis			0.7
Cystic fibrosis	2 (8.0)	3 (9.4)	
Obstructive lung disease	8 (32)	6 (19)	
Pulmonary vascular disease	0 (0)	1 (3.1)	
Restrictive lung disease	15 (60)	22 (69)	
Donor age, y	40 (28, 49)	42 (27, 49)	0.7
Donor sex			0.6
Male	16 (64)	18 (56)	
Female	9 (36)	14 (44)	
Donor type			0.7
DBD	23 (92)	28 (88)	
DCD	2 (8.0)	4 (13)	
Last EVLP PaO_2_/FiO_2_ ratio	448 (396, 500)	463 (418, 526)	0.6
EVLP duration, min	466 (376, 590)	473 (378, 607)	0.7
Total out-of-body time, min	639 (565, 785)	660 (564, 839)	0.6
**Posttransplant outcomes**			
Hospital length of stay, d	19 (13, 25)	29 (19, 40)	0.013
Ventilator support			<0.001
<48 h	15 (60)	3 (9.4)	
≥48 h to <5 d	3 (12)	7 (22)	
≥5 d	7 (28)	22 (69)	
Airway complication	5 (20)	7 (22)	0.9
Tracheostomy	5 (20)	17 (53)	0.011
90-day mortality	0 (0)	5 (16)	0.061
1-year mortality	0 (0)	8 (25)	0.007

Data are presented as median (Q1, Q3) or n (%). *p* values were calculated using the Wilcoxon rank-sum test, Pearson χ2 test, or Fisher exact test, as appropriate. DBD, donation after brain death; DCD, donation after circulatory death; EVLP, ex vivo lung perfusion; PGD3, primary graft dysfunction grade 3.

**Table 2:** End-EVLP perfusate cellular composition and blood storage duration stratified by PGD3 status.

Variable	No PGD3 n = 23	PGD3 Within 72 h n = 30	*p* Value
White blood cell count, ×10^3^/µL	1.7 ± 1.4	1.3 ± 0.7	0.13
RBC count, ×10^6^/µL	2.0 ± 0.3	1.9 ± 0.2	0.43
Platelet count, ×10^3^/µL	7.2 ± 4.7	8.8 ± 5.0	0.21
Hemoglobin, g/dL	5.8 ± 1.3	5.7 ± 0.6	0.50
Hematocrit, %	17.5 ± 3.8	16.9 ± 2.0	0.50
Storage duration of RBCs used in the EVLP perfusate, day	12.7 ± 6.7	15.3 ± 7.5	0.19

Data are presented as mean ± standard deviation. EVLP, ex vivo lung perfusion; PGD3, primary graft dysfunction grade 3; RBCs, red blood cells.

## Data Availability

The data that support the findings of this study are available from the corresponding author upon reasonable request and subject to institutional and regulatory approvals.

## Abbreviations

EVLP	ex vivo lung perfusion
FITC	fluorescein isothiocyanate
HLMVEC	human lung microvascular endothelial cell
IL	interleukin
OCS	Organ Care System
pfHb	plasma-free hemoglobin
PGD	primary graft dysfunction
PGD3	primary graft dysfunction grade 3
RBC	red blood cell
VE-cadherin	vascular endothelial cadherin
